# Oleanolic acid enhances neural stem cell migration, proliferation, and differentiation in vitro by inhibiting GSK3β activity

**DOI:** 10.1038/s41420-018-0111-0

**Published:** 2018-10-15

**Authors:** Shi Qing Zhang, Kai Li Lin, Cheuk Yu Law, Bin Liu, Xiu Qiong Fu, Wing Sze Tse, Samantha Sze Man Wong, Stephen Cho Wing Sze, Ken Kin Lam Yung

**Affiliations:** 1 Faculty of Science, Department of Biology, Hong Kong Baptist University (HKBU), Hong Kong, China; 2 HKBU Shenzhen Research Institute and Continuing Education, Shenzhen, China; 3 Guangzhou Institute of Cardiovascular Disease, The Second Affiliated Hospital of Guangzhou Medical University, Guangzhou, China; 4 Center for Cancer and Inflammation Research, School of Chinese Medicine, HKBU, Hong Kong, China

**Keywords:** Stem cells in the nervous system, Target validation

## Abstract

Oleanolic acid (OA), one of the bioactive ingredients in ginseng, has been reported to have neuroprotective activities. However, the effects and its mechanism on neural stem cell (NSC) induction are not entirely clear. In the present study, we investigated the effects of OA on promoting the migration, proliferation, and differentiation of neural stem cells (NSCs). Migration and proliferation were investigated by using neural-specific markers, neurosphere assay, and Cell Counting Kit-8, respectively. We found OA remarkably promoted neural migration and proliferation of NSCs in a time- and dose-dependent manner. Differentiation was analyzed by western blotting and immunofluorescence staining, which found MAP2 expression was remarkably increased, whereas Nestin was dramatically decreased. In addition, OA increased phosphorylation of GSK3β at Ser9 and expression of active forms of β-catenin. Furthermore, NSCs with constitutively active GSK3β (S9A) significantly suppressed the OA-induced proliferation and neural differentiation. These results showed that OA could stimulate NSC proliferation and neural differentiation in vitro via suppressing the activity of GSK3β. Our findings may have significant implications for the treatment of neurodegenerative diseases.

## Introduction

Oleanolic acid (OA), one of the key bioactive ingredients of ginseng, is a triterpenoid compound that possess pharmacological properties including neuroprotective^1^, anti-cancer, and anti-inflammatory activities^2^. Recently, OA was suggested to be a promising neuroprotective agent^3^ but its effects are far from clear. In addition, it was reported to improve neural stem cell (NSC) differentiation^4^ but the potential mechanism of OA-mediated NSC induction is unknown.

The rapidly aging population makes neurodegenerative diseases such as Alzheimer’s disease and Parkinson’s disease a primary healthcare concern. The main pathogenesis of neurodegenerative disease is the progressive loss of function or death of neurons^5^. One promising therapeutic approach for treating neurodegenerative diseases is neuron transplantation or inducing the neurogenic differentiation of NSCs in situ^6^. However, NSCs have limited ability to self-renew and differentiate into neurons, astrocytes, and oligodendrocytes under normal conditions^7^, and require induction by growth factors (GFs). It was reported that residual GFs may increase the risk of developing a tumor after transplantation^8^. As a consequence, greater attention has been paid to the development of active ingredients extracted from natural medicines that can promote NSC proliferation and neural differentiation, especially active small molecules that can effectively cross the blood–brain barrier (BBB).

GSK3β/β-catenin signaling is important in the regulation of NSC proliferation and differentiation^9^. Inactivation of GSK3β by phosphorylation at Ser9 accelerated the nuclear translocation of unphosphorylated active β-catenin, promoting NSC proliferation and differentiation^10^. These findings indicate that inactivation of GSK3β might be a promising target to induce NSC proliferation and differentiation.

In the present study, we investigated the effects of OA on NSC proliferation and neural differentiation. Considering the importance of the activated state of GSK3β in controlling NSC induction, we used genome-editing techniques to investigate the role of GSK3β activation in regulating NSC proliferation and differentiation. To the best of our knowledge, this is the first study to confirm that OA mediates NSC proliferation and neural differentiation through suppressing the activation of GSK3β.

## Results

### OA increased NSC migration

**Fig. 1:**
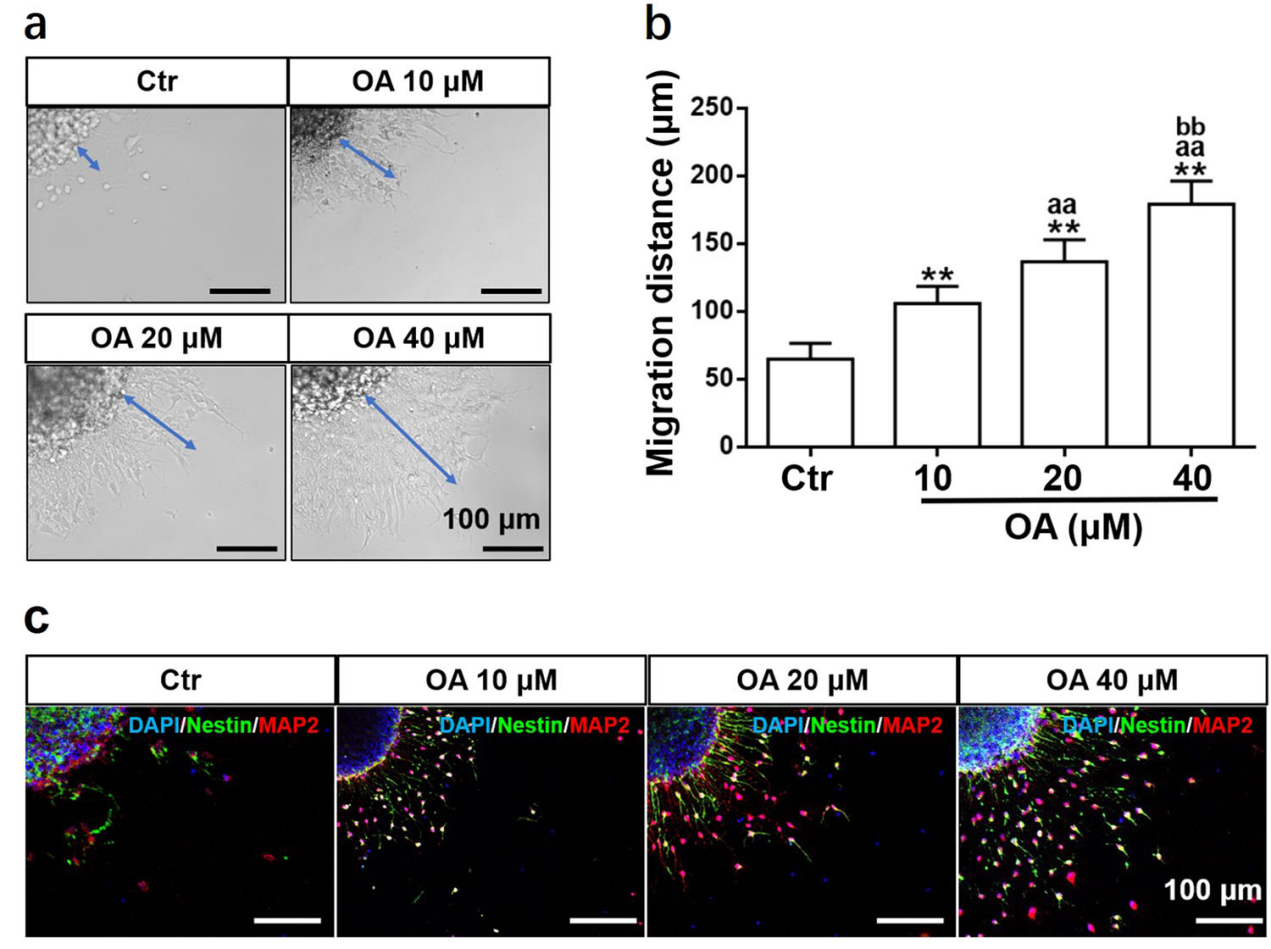
OA increases NSC migration in the neurosphere assay. **a** Neurospheres seeded onto a 24-well PDL-coated plate were treated with different concentrations of OA for 24 h. Blue arrows indicate the migration distance from neurospheres. **b** OA-induced migration distance increased in a dose-dependent manner. Data are presented as means ± SD, *n* = 5. **p < 0.01, compared with the Ctr group; ^aa^*p* *<* 0.01, compared with 10 μM OA group; ^bb^*p* *<* 0.01, compared with 20 μM OA group. **c** Migrating cells expressed Nestin and MAP2 neural markers. Scale bars: 100 μm

Migration of NSCs was analyzed after treatment with OA (10, 20, and 40 μM). The neural cells were observed to emerge from the neurosphere and migrate along a radial axis (Fig. 1). The OA treatment significantly increased the migration distance in a dose-dependent manner (Fig. 1a, b). The immunofluorescence staining showed the cells derived from the neurospheres were MAP2-positive and mostly Nestin-positive cells (Fig. 1c).

### OA promoted proliferation of NSCs

**Fig. 2:**
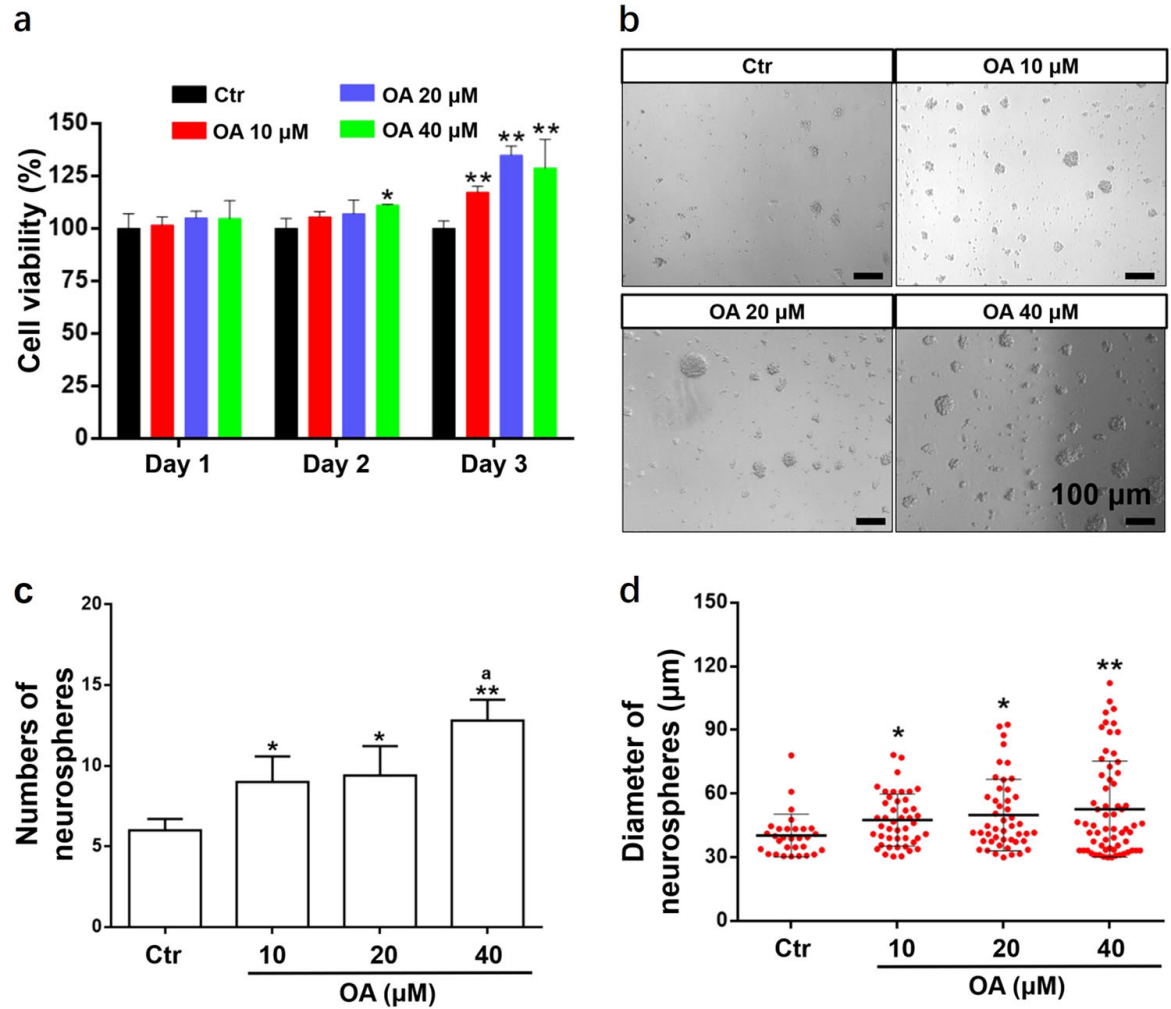
OA promotes the proliferation of NSCs. NSCs were treated with different concentrations of OA (10, 20, and 40 μM). OA-mediated NSC proliferation was analyzed by CCK-8 assay after OA treatment for 1, 2, or 3 days (**a**) and neurosphere assay was performed after OA treatment for 3 days (**b**–**d**). **a** CCK-8 assay showed cell viability was significantly enhanced in a dose- and time-dependent manner. Data are presented as means ± SD, *n* = 5. **b** An image of neurospheres after treatment with OA. Scale bars: 100 μm. The number (**c**) and the diameter (**d**) of neurospheres (diameter > 30 μm) increased with OA treatment in a dose-dependent manner. Data are presented as mean ± SD, *n* = 5. *p < 0.05 and **p < 0.01, compared with the Ctr group; ^a^*p* *<* 0.05, compared with 10 μM OA group

Proliferation of NSCs induced by OA was investigated by CCK-8 assay and neurosphere assay. The CCK-8 assay showed a slight absorbance increase on day 2 after culture, but this was not significant. On day 3 after treatment, the absorbance increased steeply and cell viability in the 20 and 40 μM OA groups was significantly higher than in the 10 μM OA group (Fig. 2a). The cell proliferation and cell viability increased significantly with OA treatment in a dose-dependent manner. The neurosphere assay showed the number and diameter of neurospheres generated from NSCs were significantly increased with the OA treatment in a dose-dependent manner (Fig. 2b–d). These results indicate that OA can significantly promote NSC proliferation and self-renewal in vitro.

### OA promoted the neural differentiation of NSCs

**Fig. 3:**
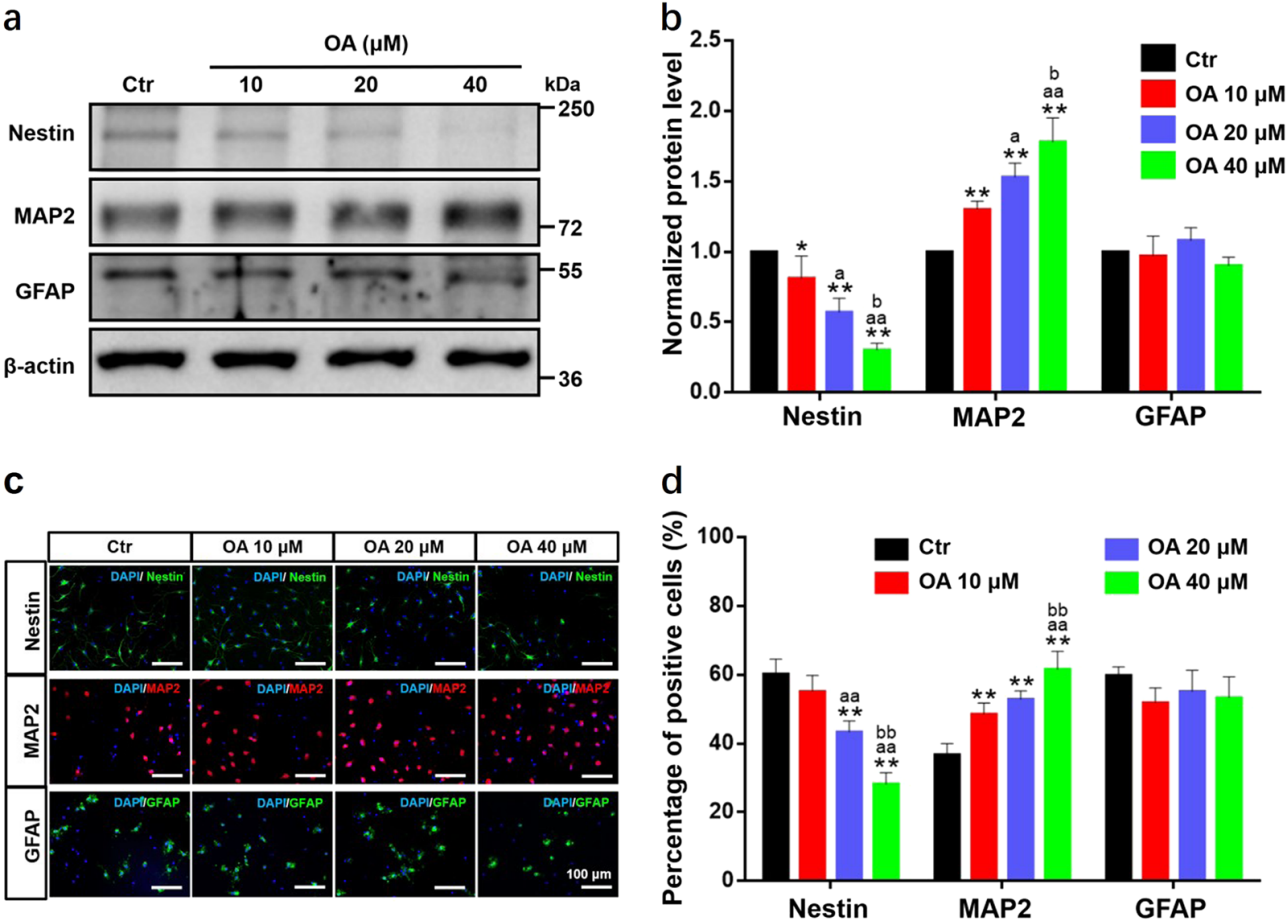
OA selectively induces neural differentiation of NSCs. Treatment with OA for 7 days dose-dependently promoted the neural differentiation of NSCs, but suppressed astrocyte differentiation, as determined by western blotting (**a**, **b**) and immunofluorescence staining (**c**, **d**). **a**, **b** OA significantly and dose-dependently decreased the expression of Nestin and increased the expression of MAP2, but expression of GFAP was not significantly affected. **c**, **d** OA significantly and dose-dependently decreased the proportion of Nestin-positive cells and increased the proportion of MAP2-positive cells. Scale bars: 100 μm. All data are presented as means ± SD from three independent experiments. *p < 0.05 and **p < 0.01, compared with the Ctr group; ^a^*p* *<* 0.05 and ^aa^*p* *<* 0.01, compared with 10 μM OA group; ^bb^*p* *<* 0.01, compared with 20 μM OA group

NSCs have the potential to differentiate into multiple neural lineages, mostly neurons and astrocytes. Differentiation of NSCs after treatment with OA was determined by measuring the levels of three neural markers: Nestin, MAP2, and GFAP. The western blot analyses showed OA significantly and dose-dependently reduced the expression of Nestin and upregulated the expression of MAP2, which is an exclusive dendritic protein in neurons. Furthermore, there was a decreasing trend in the expression of GFAP after treatment with 20 and 40 μM OA (Fig. 3a, b). These results demonstrate that OA can markedly promote neural differentiation of NSCs and suppress astrocyte differentiation in a dose-dependent manner. These findings were also confirmed by immunofluorescence staining. As shown in Fig. 3c, d, the percentage of MAP2-positive cells significantly increased with increasing OA concentration, whereas the percentage of GFAP-positive cells showed a downward trend. Interestingly, we observed many MAP2 and Nestin double-stained cells in the immunofluorescence staining, indicating some of the NSC-derived neurons were approaching maturation^11^.

### OA directly targeted GSK3β

**Fig. 4:**
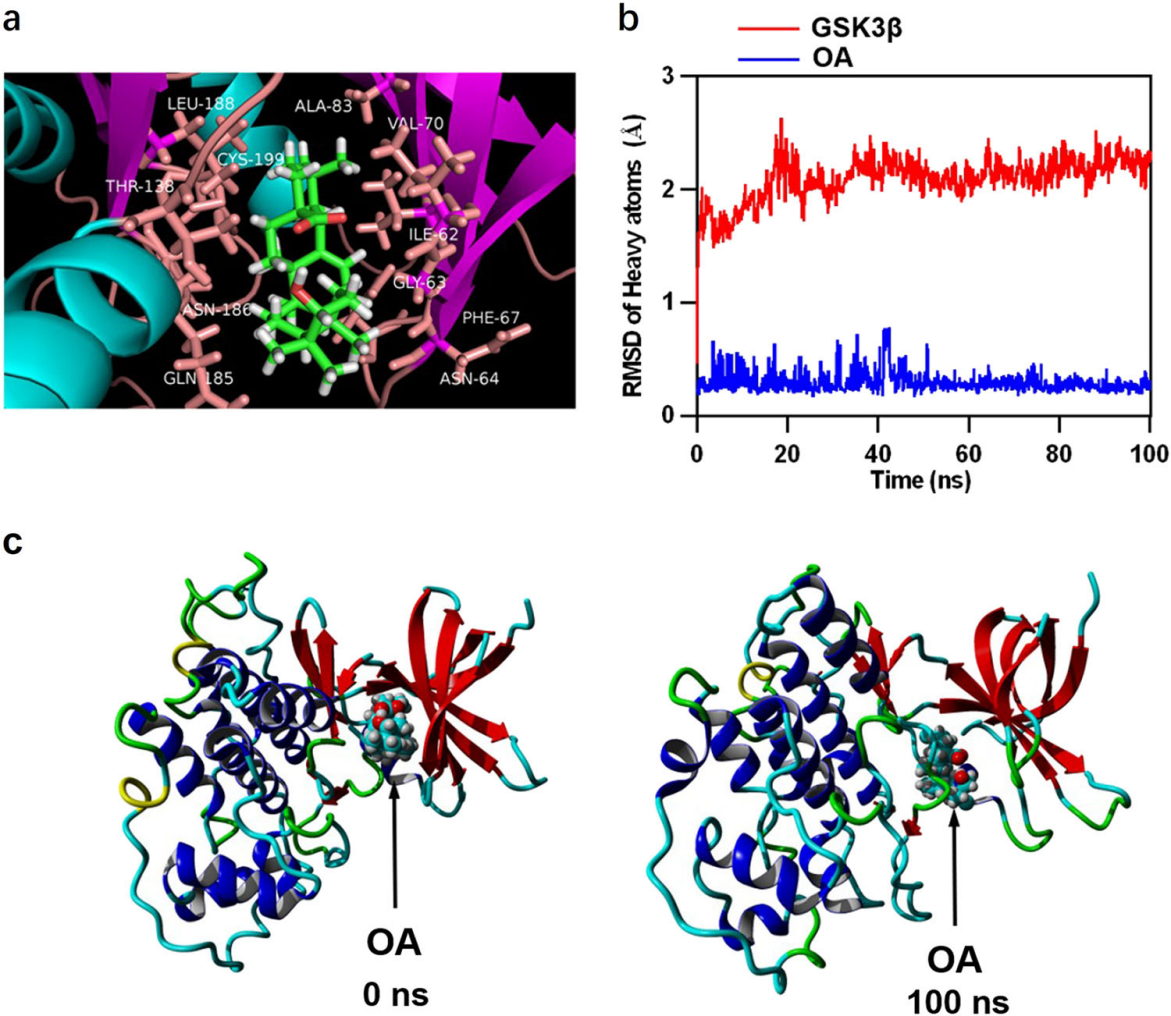
Molecular docking and molecular dynamics simulation. **a** Three-dimensional crystal structure of the complex of OA (ZINC95098891) with GSK3β (PDB ID: 4ACC). **b** Plots of root-mean-square deviation (RMSD) of heavy atoms of GSK3β (red) and OA (blue). **c** Surface presentation of the GSK3β-OA complex crystal structure at 0 and 100 ns

The GSK3β/β-catenin signaling is considered to be a critical regulator for NSC proliferation and differentiation^9^. Inhibition of GSK3β activity by phosphorylation at Ser9^12^ causes unphosphorylated active β-catenin to accumulate in the cytoplasm and then translocate into the nucleus, promoting NSC proliferation and differentiation^10^. To explore the interaction between OA and GSK3β, we performed a molecular docking analysis using the Autodock Vina molecular docking program. The binding energy of the GSK3β-OA complex was − 8.871 kcal/mol, which suggested good binding ability. The three-dimensional binding conformation of the GSK3β-OA complex showed that OA interacted with GSK3β at LEU-188, THR-138, ASN-186, GLN-185, CYS-199, ALA-83, VAL-70, ILE-62, GLY-63, PHE-67, and ASN-64 via van der Waals force (Fig. 4a). To further verify the molecular docking results, the best conformation of GSK3β-OA was taken as the starting conformation for the MD simulation by YASARA. As shown in Fig. 4b, the heavy atoms root-mean-square deviation (RMSD) track of the GSK3β (Fig. 4b, red line) mildly fluctuated around 2 Å over 0–100 ns and the RMSD track of OA (Fig. 4b, blue line) fluctuated around 0.2 Å during the MD simulation. The surface visualization models of the GSK3β–OA complex at 0 and 100 ns are shown in Fig. 4c. We found OA was stably presented at the center of the GSK3β binding site throughout the MD simulation. These results indicate that the binding between GSK3β and OA is stable, and that OA likely targets GSK3β directly.

### OA-mediated NSC induction through suppressing the activation of GSK3β

**Fig. 5:**
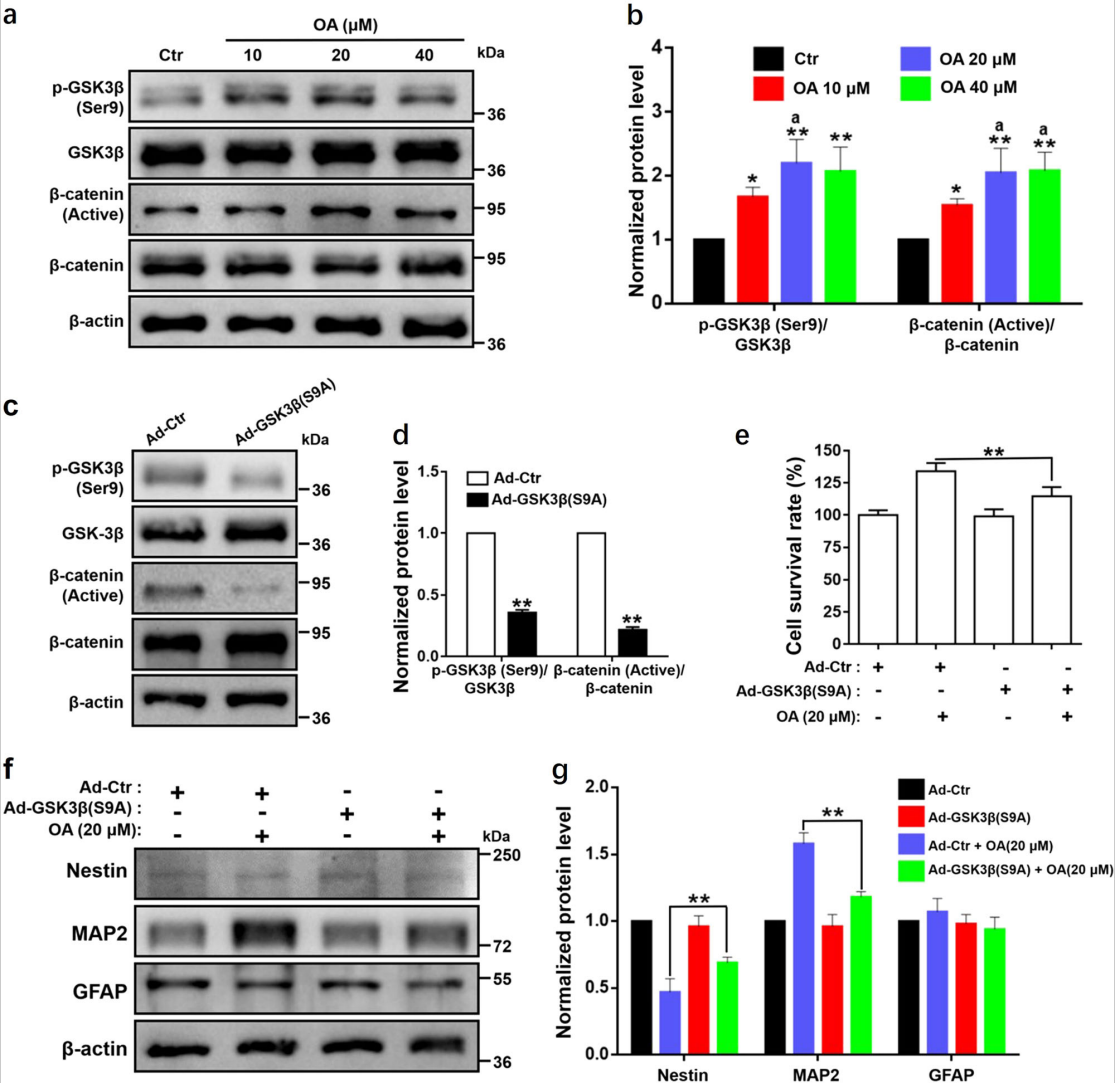
OA regulates the activation of GSK3β in NSC induction. Proliferation and differentiation of NSCs mediated by OA depends on the suppressing of the activation of GSK3β. **a** Western blot analyses and **b** relative optical density of diverse pathway markers including β-catenin (active), β-catenin, p-GSK3β (Ser9), and GSK3β in NSCs after treatment with OA for 7 days. *p < 0.05 and **p < 0.01, compared with the Ctr group; ^a^*p* *<* 0.05, compared with 10 μM OA group. **c** Western blot analyses and **d** relative optical density of p-GSK3β (Ser9), GSK3β, p-GSK3β (Ser9), and GSK3β in NSCs after transfection with GSK3β (S9A) adenovirus for 2 days. **p < 0.01, compared with the Ad-Ctr group. **e** NSCs with GSK3β (S9A) significantly suppressed cell viability, as examined by CCK-8 assay. **p < 0.01, compared with Ad-Ctr + 20 μM OA group. **f** Western blot analyses and **g** relative optical density of diverse differentiation markers including Nestin, MAP2, and GFAP in NSCs transfected with GSK3β (S9A) treated with OA (20 μM). NSCs with GSK3β (S9A) significantly suppressed NSC neural differentiation. **p < 0.01, compared with Ad-Ctr + 20 μM OA group. All data are presented as means ± SD from three independent experiments

According to the results of the western blot analysis, NSCs treated with OA for 7 days showed significant and concentration-dependent increases in the expression ratios of p-GSK3β (Ser9) to GSK3β and of β-catenin (active) to β-catenin compared with the control group (Fig. 5a, b). The results showed that OA-mediated NSC proliferation and differentiation might depend on the activation of GSK3β/β-catenin pathway.

To further confirm the involvement of the activation of GSK3β in OA-mediated NSC proliferation and differentiation, we activate GSK3β by transfection of constitutively active GSK3β mutant at S9A using the GSK3β (S9A) adenovirus. The transfection was confirmed by western blot analysis (Fig. 5c, d). The expression ratio of p-GSK3β (Ser9) to GSK3β was significantly decreased to about 30% in GSK3β (S9A) small interfering RNA (siRNA) group compared with the control siRNA group, leading to significant downregulation of the ratio of β-catenin (active) to β-catenin. As expected, cell viability of NSCs^GSK3β (S9A)^ induced by OA was significantly attenuated (Fig. 5e). Furthermore, OA-mediated NSCs mutated with GSK3β (S9A) had significantly increased Nestin expression, but decreased MAP2 expression (Fig. 5f, g). Overexpression of constitutively active GSK3β (S9A) significantly attenuated OA-mediated NSC proliferation and differentiation. All these results suggest that the activated state of GSK3β is critical in OA-mediated proliferation and differentiation of NSCs.

## Discussion

As neurodegenerative diseases are typically characterized by the gradual loss of structure and/or function of neurons in the brain, neuron replacement has become the most promising method to treat neurodegenerative diseases^13^. In the present study, we investigated OA, a bioactive molecule, as a potential treatment for neurodegenerative diseases through the induction of NSCs. We confirmed that OA could dose-dependently promote the migration and proliferation of NSCs. In addition, OA could dose-dependently induce NSC neural differentiation, while suppressing astrocyte differentiation. Many macromolecular drugs cannot pass the BBB to target pathological changes in the brain^14^. Meanwhile, a study demonstrated that OA could be effectively delivered across the BBB^15^, further supporting the development of OA as a promising drug for treating neurodegenerative diseases that can be administered orally.

Recently, several NSC replacement therapies have been rapidly developed that may be promising treatments for neurodegenerative diseases, but they require in vitro induction of NSCs into functional neurons. NSCs have limited ability to proliferate and differentiate in natural conditions and require induction by GFs^16^ such as EGF and bFGF. However, in cell replacement therapy, GFs may lead to a higher risk for carcinogenicity in vivo after transplantation^17,18^. As a natural active component, OA might be an ideal alternative for GFs to induced NSC proliferation and differentiation efficiently and safely.

GSK3β has a critical role in regulating the behavior and fate of NSCs. Inhibiting the activity of GSK3β can significantly promote NSC proliferation and neural differentiation^19^. We investigated the ability of OA to target GSK3β directly by performing molecular docking and MD simulations to assess the mechanism by which OA and GSK3β interact. The binding energy of the OA-GSK3β complex indicated strong binding interactions. Moreover, OA binding to GSK3β was through LEU-188, THR-138, ASN-186, GLN-185, CYS-199, ALA-83, VAL-70, ILE-62, GLY-63, PHE-67, and ASN-64 via van der Waals force. These residues are key residues involved in inhibiting the binding domain of GSK3β^20^. Furthermore, the MD simulations demonstrated that the binding conformation of OA-GSK3β was stable. These findings indicated that OA directly targets GSK3β and can potentially act as a GSK3β inhibitor. This was consistent with the Western blotting results that showed OA significantly and dose-dependently suppressed the activation of GSK3β. To further confirm that the effect of OA on NSC induction was mediated through GSK3β, we transfected NSCs with the constitutively active GSK3β (S9A) adenovirus. Western blotting showed successful knockdown of p-GSK3β (Ser9), leading to downregulation of the expression of β-catenin (active). The proliferation and differentiation mediated by OA were significantly restrained in NSCs transfected with GSK3β (S9A) adenovirus. These results suggest the activity of GSK3β might be critical in OA-mediated NSC proliferation and differentiation. In this study, the change of activated state of GSK3β made corresponding change of β-catenin (active). Both GSK3β and β-catenin (active) are regulated by multiple pathways, one of which is Wnt signaling pathway which plays a crucial role in NSC proliferation and differentiation^21^. Although further research is needed to fully explore the role of Wnt signaling pathway involved in OA-mediated NSC induction.

NSCs can be an essential source of neural cells for the treatment of many incurable diseases including neurodegenerative diseases. One of the major challenges is to induce neural differentiation of NSCs over other lineages and to ensure the treatment is safe. In this study, we demonstrated that OA promoted the differentiation of NSCs into neurons but suppressed the formation of astrocytes. OA is derived from ginseng, which has a long history in many clinical applications, and may provide a safe and efficient way for NSC induction. Our significant findings hold much promise for improving NSC replacement therapy in neurodegenerative diseases and other related diseases.

## Methods and materials

### NSC isolation and primary cell culture

The study was approved by the Department of Health, the Government of the HKSAR. The modified experimental protocol^22^ was carried out in accordance with relevant guidelines and regulations of the Animal Ethics Committee at HKBU. Rats (Sprague–Dawley) at postnatal day 1 to 2 were purchased from the Chinese University of Hong Kong. The subventricular zone of the brain was dissected out and manually cut into pieces. After digestion in trypsin at 37 °C for 15 min, the tissue suspension was filtered through a Cell Strainer with a 40 µm mesh. The filtrate was centrifuged for 5 min at 200 × *g* and the supernatant was discarded. The pellet was resuspended in neurobasal medium which contains 20 ng/mL epidermal GF (EGF, PeproTech, Rocky Hill, NJ, USA), 20 ng/mL basic fibroblastic GF (bFGF, PeproTech), 1% PSN (Thermo, Waltham, MA USA), and 2% B27 supplement (Thermo), and then cultured at 37 °C in a 5% CO_2_ humidified incubator. Half of the medium was changed every 2–3 days. The cells were passaged by mechanical method when the diameter of the neurospheres reached 150–200 μm.

### Migration analysis

OA (purity assayed by high-performance liquid chromatography: 98.33%) was purchased from Must Bio-Technology Co., Ltd (Chengdu, China). Neurospheres (around 200 μm in diameter) were seeded on coverslips, which had been coated with poly-d-lysine (PDL; Sigma, St. Louis, MO, USA) for 30 min to enable neurospheres to attach. The neurospheres were exposed to a series of OA concentrations (10, 20, and 40 μM). After incubation for 24 h, photos of the neurospheres were taken under an inverted microscope. The migration distance of neural cells from the edge of the neurospheres was measured by Image-J software. Immunostaining was performed to visualize Nestin (NSCs marker)^23^ and microtubule-associated protein-2 (MAP2, neuron marker)^24^ as described below.

### Neurosphere assay

Single cells were dissociated from the neurospheres and adjusted to a density of 2.5 × 10^4^ cells/mL. The cells were seeded onto a 96-well plate (200 μL/well) and treated with various concentrations of OA (10, 20, and 40 μM) without GFs. After 3 days of incubation, the number and diameter of neurospheres (diameter > 30 μm^25^) were analyzed by Image-J software.

### CCK-8 assay

Single cells were dissociated from the neurospheres and adjusted to a density of 2.5 × 10^4^ cells/mL. The cells were seeded onto a 96-well plate (200 μL/well) and treated with various concentrations of OA (10, 20, and 40 μM) for different experimental periods (1, 2, and 3 days). At the end of each experimental period, CCK-8 (DOJINDO, Rockville, MD, USA) reagent was added to measure cell proliferation. After further incubation at 37 °C for 4 h, the absorbance of each well was measured at a wavelength of 450 nm using an automatic microplate reader (BioTek).

### Cell differentiation study

Single cells were seeded at 2 × 10^5^ cells/well onto a six-well PDL-coated plate and then treated with different concentrations of OA (10, 20, and 40 μM). After incubation for 7 days, NSC differentiation was detected by measuring the levels of differentiation markers, including Nestin, MAP2, and glial fibrillary acidic protein (GFAP, astrocytes marker)^24^, by western blotting and immunofluorescence staining.

### Immunofluorescence staining

Cells were fixed with 4% paraformaldehyde (Sigma) for 30 min at room temperature. The cells were then incubated with the primary antibodies including anti-Nestin antibody, anti-MAP2 antibody, and anti-GFAP antibody (1:1000, Millipore, Temecula, CA, USA) in phosphate-buffered saline (PBS), with 0.1% Triton X-100 (Sigma-Aldrich) and 2% normal goat serum (Vector Laboratories, Burlingame, CA, USA) overnight at 4 °C. After rinsing with PBS three times, the cells were incubated with the secondary antibodies at room temperature for 3 h. The cells were then incubated with 1 μg/mL 4′,6-diamidino-2-phenylindole (Roche, Switzerland) for 15 min to stain the nuclei. After rinsing with PBS, fluorescence mounting medium (Dako, Aglient, Santa Clara, CA, USA) was added, and immunoreactivity images were obtained and processed under a confocal microscope (FluoView FV1000, Olympus, Tokyo, Japan).

### Western blotting

Cellular proteins were extracted using protein extraction reagent (Novagen, Madison, WI, USA) supplemented with a protease inhibitor cocktail (Calbiochem, San Diego, CA, USA) on ice. Protein concentration was determined with a protein assay kit (Bio-Rad, Hercules, CA, USA). Next, proteins (30 μg) were separated on a 10% SDS-polyacrylamide gel and transferred to a polyvinylidene difluoride membrane (Bio-Rad). The membrane was incubated with specific primary antibodies overnight at 4 °C. Primary antibodies included anti-Nestin antibody (1:1000, Millipore), anti-MAP2 antibody (1:1000, Millipore), anti-GFAP antibody (1:1000, Millipore), anti-p-GSK3β (Ser9) antibody (1:1000, Cell Signaling, Beverly, MA, USA), anti-GSK3β antibody (1:1000, Cell Signaling), anti-Non-p (active) β-catenin antibody (1:1000, Cell Signaling), and anti-β-catenin antibody (1:1000, Cell Signaling). The membrane was then incubated with the secondary antibody for 1 h at room temperature. β-Actin antibodies (1:5000, Sigma) were used as the reference. The bands were visualized and imaged using the ChemiDoc Touch imaging system (Bio-Rad).

### Molecular simulation of the interaction of OA and GSK3β

Autodock Vina molecular docking software (Scripps Research Institute, La Jolla, CA, USA)^26^ was used to analyze the binding mechanism between GSK3β (PDB ID: 4ACC) and OA (ZINC95098891). YASARA was used to perform the MD simulation^27^ and the energy minimization of the ligands, and AMBER 03 forcefield was used to run all simulations. The star conformation was considered the best conformation for the molecular dynamics (MD) simulation. In this study, the inhibitor-binding domain of GSK3β corresponded to the protein-ligand binding site of GSK3β. The protein structure of GSK3β was prepared by removing water molecules and bound ligands. Briefly, for the solvation of the receptor–ligand complex, 0.9% NaCl was placed in a dodecahedron box, with a distance of 5 Å between the box and solute. The simulated annealing minimizations were initially set at 298 K and velocities were scaled down by 0.9 with every ten steps lasting for 5 ps. After the energy was minimized, the temperature of the system was adjusted using a Berendsen thermostat to minimize temperature control influences. In addition, velocities were rescaled only every 100 simulation steps, whenever the mean of last 100 detected temperatures converged. Finally, 100 ns MD simulations were conducted every 2 fs and the coordinates of the complexes were saved every 10 ps.

### Adenovirus-mediated expression of constitutively active GSK3β

The pAdM-FH-GFP-GSK3β (S9A) adenoviral vector [Ad-GSK3β (S9A)] expressing the constitutively active GSK3β with the serine residue at position 9 mutated to alanine [GSK3β (S9A)] and the empty adenoviral vector were generated by Vigene Bioscience (Rockville, MD, USA). Single cells dissociated from neurospheres were seeded at 2 × 10^5^ cells/well onto a six-well PDL-coated plate. The NSCs were transfected with Ad-GSK3β (S9A) or empty adenoviral vector (Ad-Ctr) as the control at a multiplicity of infection of 40 in the presence of 6 μg/mL polybrene (Sigma). The medium containing the virus was then exchanged for fresh medium after 12 h of transfection. Cells were cultured for at least 48 h after transfection before further experiments were performed. The transgene expressions were confirmed in the infected cells by analyzing protein expressions.

### Statistical analysis

All data were expressed as means ± SD. Statistical analysis was performed by one-way analysis of variance. Relative risk was expressed as odds ratios with 95% confidence interval and statistical significance was defined as *p* < 0.05. The statistical analyses were conducted using GraphPad Prism.
