# Correction: Oleanolic acid enhances neural stem cell migration, proliferation and differentiation in vitro by inhibiting GSK3β activity

**DOI:** 10.1038/s41420-021-00565-2

**Published:** 2021-10-21

**Authors:** Shi Qing Zhang, Kai Li Lin, Cheuk Yu Law, Bin Liu, Xiu Qiong Fu, Wing Sze Tse, Samantha Sze Man Wong, Stephen Cho Wing Sze, Ken Kin Lam Yung

**Affiliations:** 1 Faculty of Science, Department of Biology, Hong Kong Baptist University (HKBU), Hong Kong, China; 2 HKBU Shenzhen Research Institute and Continuing Education, Shenzhen, China; 3 Guangzhou Institute of Cardiovascular Disease, The Second Affiliated Hospital of Guangzhou Medical University, Guangzhou, China; 4 Center for Cancer and Inflammation Research, School of Chinese Medicine, HKBU, Hong Kong, China

**Keywords:** Stem cells in the nervous system, Target validation

Correction to: *Cell Death Discovery* 10.1038/s41420-018-0111-0, published online 15 October 2018

The original version of this article unfortunately contained errors in Fig. 3c. The immunofluorescence images of Nestin and MAP2 were accidentally used in Ctr and OA 20 μM groups. The corrected images are provided below. The authors confirm that these errors do not affect the scientific conclusions of the article and apologize for any inconvenience caused.

**Figure:**
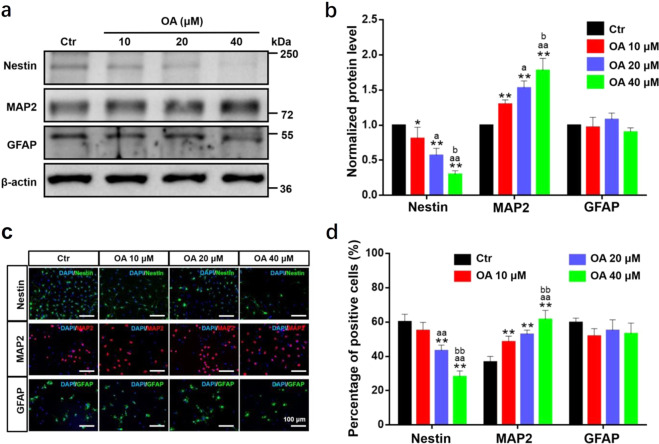
Fig. 3.

